# *policedatR*: a comprehensive R package for stop and search data in England and Wales

**DOI:** 10.1186/s40163-025-00266-6

**Published:** 2026-01-28

**Authors:** Jolyon Miles-Wilson, Celestin Okoroji

**Affiliations:** 1 Just Knowledge Community Interest Company, 167-169 Great Portland Street, London W1W 5PF, UK; 2 London School of Economics and Political Science, Houghton St, London WC2A 2AE, UK

**Keywords:** Stop and search, Police, Disproportionality, R, Racial disparities, Accountability

## Abstract

Research on Stop and Search in England and Wales is constrained by substantial barriers to data access, inconsistent geographic coverage, and technical complexity. This paper presents *policedatR*, an R package that addresses these challenges by providing streamlined access to comprehensive stop and search data from the data.police.uk Application Programming Interface (API). *policedatR* automates data acquisition across multiple geographic scales, enriches datasets with population estimates and geographic identifiers, and includes functions for analysing the data, including calculating ethnic disproportionality. We describe the architecture and main functionalities of *policedatR* and demonstrate its capabilities and utility with analyses of temporal trends, geographic variation and ethnic disparities at national (e.g. countrywide, Police Force Area) and local (e.g. sub-local authority) levels. We also provide an example of how data acquired using the package can be harmonised with other datasets (in this case the English Indices of Deprivation) to explore broader questions on stop and search and society. By transforming thousands of individual API calls into a straightforward analytical workflow, *policedatR* facilitates rigorous empirical research and *supports* democratic accountability in policing.

## Introduction

‘Stop and Search’ is an investigative police power in the United Kingdom (UK)^1^, authorised under various legislation, that enables officers to temporarily detain, question and search individuals or vehicles. Stop and search has come under sustained public interest because it restricts the liberty of a person without charge and at the discretion of police officers, often based on ‘gut feeling’ (Flacks, 2018), and in particular because it is differentially applied across social groups, especially against Black people (Agnew-Pauley et al., 2025; Eastwood et al., 2013; Ffrench, 2021; Shiner et al., 2018). Disproportionate and oppressive uses of stop and search against Black communities have been implicated in a range of significant incidents of social unrest and examined in several subsequent or related enquiries and reviews (Ariel & Tankebe, 2018; Lammy, 2017; Macpherson, 1999; Scarman, 1986).

Despite the political and social importance of stop and search and the need for public accountability, the available sources of stop and search data in England and Wales have significant limitations that serve as obstacles to empirical enquiry and public understanding. Consequently, a substantial proportion of empirical stop and search studies are based in the United States (Agnew-Pauley et al., 2025), where the similar but distinct legal concept of ‘stop and frisk’ is deployed. The ecological relevance of US studies to England and Wales is limited given the considerable differences in societal, legal, and policing contexts, therefore empirical work from the US cannot fully compensate for the limited empirical insights for England and Wales.

The sparsity of research for the English and Welsh context may be partially attributable to data availability. In England and Wales, stop and search data is typically acquired through annual official data releases (*Police Powers and Procedures England and Wales Statistics*, 2025). Borooah(2011) argues that “the public debate in Britain on the incidence and consequences of disparity in stops has taken place entirely in the context of data published by the MoJ” (2011, p. 460). Nevertheless, data may also be obtained via Freedom of Information (FOI) requests, direct contacts with police forces and through data downloads from police.uk,^2^ the latter of which we consider to be the most comprehensive. Each of these modes of data acquisition has limitations which can make it cumbersome for research purposes, namely issues with timeliness, legislative scope, geographic coverage and specificity. In this section we provide an over-view of these data sources.

‘Police Powers and Procedures’ (PPaP) is an annual data release from the Home Office, which covers searches under Section 1 of the Police and Criminal Evidence Act (PACE) 1984, Section 60 of the Criminal Justice and Public Order Act (CJPOA) 1994 and Sections 44 and 47A of the Terrorism Act 2000. These data are the most commonly used for quantitative research on stop and search and contain simple counts of the number of searches by demographic variables (e.g. ethnicity) and resultant outcomes (e.g. arrest).

In a seminal report making use of PPaP data (2012), Eastwood et al. (2013) identified drug searches as a key driver of ethnic inequality. They showed that Black people were 6.3 times more likely to be stopped and searched for drugs in England and Wales than White people. Shiner and colleagues (2018) made extensive use of a later release of the same data (2017). Their report showed that stop and search was increasingly concentrated on drug offences (two-thirds of stops at the time of publication) and that Black people were stopped and searched at eight times the rate of White people.

While these contributions are important, the analyses are limited by the coverage of the data release, whose variables are determined by data providers and out of the control of the researcher. For example, PPaP groups the legislation used to justify stops; those made under the Misuse of Drugs Act 1971 are grouped in the same category as those made under PACE, making it impossible to separate the two powers. In addition, decisions on data structure may vary between years. For instance, in the 2018 PPaP release, the breakdown of stops by ethnicity included stops made under the Terrorism Act, whereas the 2017 release did not, creating obstacles to accurate comparisons across releases.

The timeliness of PPaP also poses challenges. The most recent release was September 2024, and this covered stop and search records for the 12 months to March 2024. Thus, by the time of release the data were already five months out of date. Factoring in the time required to conduct research and publish findings means that insights into stop and search based on these data are often at least 12 months out of date, reducing their relevance to public discourse and decision-making.

Finally, PPaP data is organised by Police Force Area (PFA), with no other geographic specification possible. Although this is a useful unit for administrative enquiry, the low geographic resolution makes it impossible to investigate questions pertaining to the nuance of policing space, which is known to be critically important, and by some views the fundamental objective, of stop and search (Flacks, 2020; Shiner et al., 2018). These limitations restrict researchers’ ability to conduct timely, granular analyses of stop and search practices, ultimately constraining the depth and responsiveness of empirical research needed to inform policy decisions and public discourse.

Another method of acquiring stop and search data is via FOI requests. FOIs are the least efficient method for obtaining data. Since much stop and search data is available by other means, most researchers will not need to use FOI. However, there are some instances where information not presently in the public domain is desired, for example, a breakdown of stop and search activity by legislation for smaller geographic areas such as postcode.

FOI requests do not guarantee access to the requested information, as data holders retain discretionary authority over release decisions. Requests frequently yield partial or no data and can be considerably time and resource intensive for all parties (see Ashby & and Tompson, 2017). Eastwood et al. (2013) sought ethnic breakdowns of drug-related stops resulting in disposals such as cannabis warnings. Only 28 of 42 forces responded and did so with variable data quality. Consequently, the analysis was limited to Metropolitan Police data. StopWatch’s investigation of stop and search practices on under-18s in 2013/14 received responses from only 18 of 43 forces, with Flacks (2018) noting variable data quality, particularly regarding ethnicity recording. The data revealed that these 18 forces stopped a total of 99,402 under-18s, of which 10,808 (11%) were arrested. However, these results were based on less than half of the forces in England and Wales, and this exemplifies how relying on FOI requests for stop and search data can impede research.

Another source of stop and search data is the open-source data provided on police.uk (Quinn et al., 2019).^3^ The website was established in 2008 and provides comprehensive stop and search data for all police forces from December 2014 (Braakmann, 2022). The data are provided at person level and include the date and time of the search, demographic information about the person searched, the legislation used, the object and outcome of the search, and longitude and latitude information.

These data have been used in several recent studies. Braakmann (2022) found that increased stop and search following a murder had little effect on reducing crime, and Suss and Oliveira (2022) found that, in London, economic inequality based on housing values predicts the incidence of stop and search at Lower Super Output Area (LSOA) level.

These studies demonstrate the utility of the data provided on police.uk. The granularity of these data allows more nuanced questions to be interrogated, including the spatial and temporal characteristics of stop and search. However, to make best use of these data some level of proficiency with computer programming, data carpentry (Teal et al., 2015) and Geographic Information Systems (GIS) is required. In addition, data downloads from police.uk are separated by PFA whereby each PFA is provided as a separate file for each month of data. Downloading one year of data for all 43 police forces therefore requires downloading 516 separate files, which then need to be joined. These facts mean that there are considerable time and skill barriers in working with these data.

In this paper we present a solution to the problem of stop and search data acquisition in the form of an R Package (‘*policedatR*’) that retrieves and processes publicly available stop and search data into a reproducible format readily usable for analysis.^4^ The *policedatR* package allows users to programmatically access data from data. police.uk and parse them into official geographic units, merge them with area- and ethnicity-specific population estimates from Census 2021, and to quickly produce useful summary insights, including estimates of racial disproportionality. *policedatR* thus makes it simpler and quicker than was previously possible for researchers to acquire and analyse data on stop and search. In the remainder of this paper, we describe the architecture of *policedatR* and provide example analyses showing how it can be used to acquire and analyse data, and how data acquired using the package can be combined with other data sources.

## Methods

The data.police.uk site offers two access methods: manual datafile downloads by PFA and time period, or HTTP requests via the Application Programming Interface (API). Users interested in only a handful of PFAs and/or time periods may find the datafile download option suitable for their needs. However, in cases where data are desired for multiple PFAs and/or time periods, downloading datafiles and manually combining them quickly becomes inefficient. In addition, it is often desirable to organise the data by other units of geography relevant to variations in ecological factors. This can be achieved using HTTP requests to the API to specify bespoke areas for which to acquire stop and search records. There are challenges with this approach too, since it requires familiarity with APIs and GIS, as well as data science skills to compile the results into a single dataset.

There are a handful of open resources that facilitate interaction with the data.police.uk API. To our knowledge, the most developed of these is ‘ukpolice’ (Odell & Tierney, 2020) which allows R users to make HTTP requests to the data.police.uk API and outputs the results as data frames containing the requested data.

At the time of writing, ‘ukpolice’ does not acquire data by administrative or statistical areas such as local authorities or Census geographies, or for combining data from multiple requests to create a single dataset for comparative analysis. These are desirable functionalities because they make it possible to explore geographic and administrative variations in stop and search across England and Wales.

**Fig. 1:**
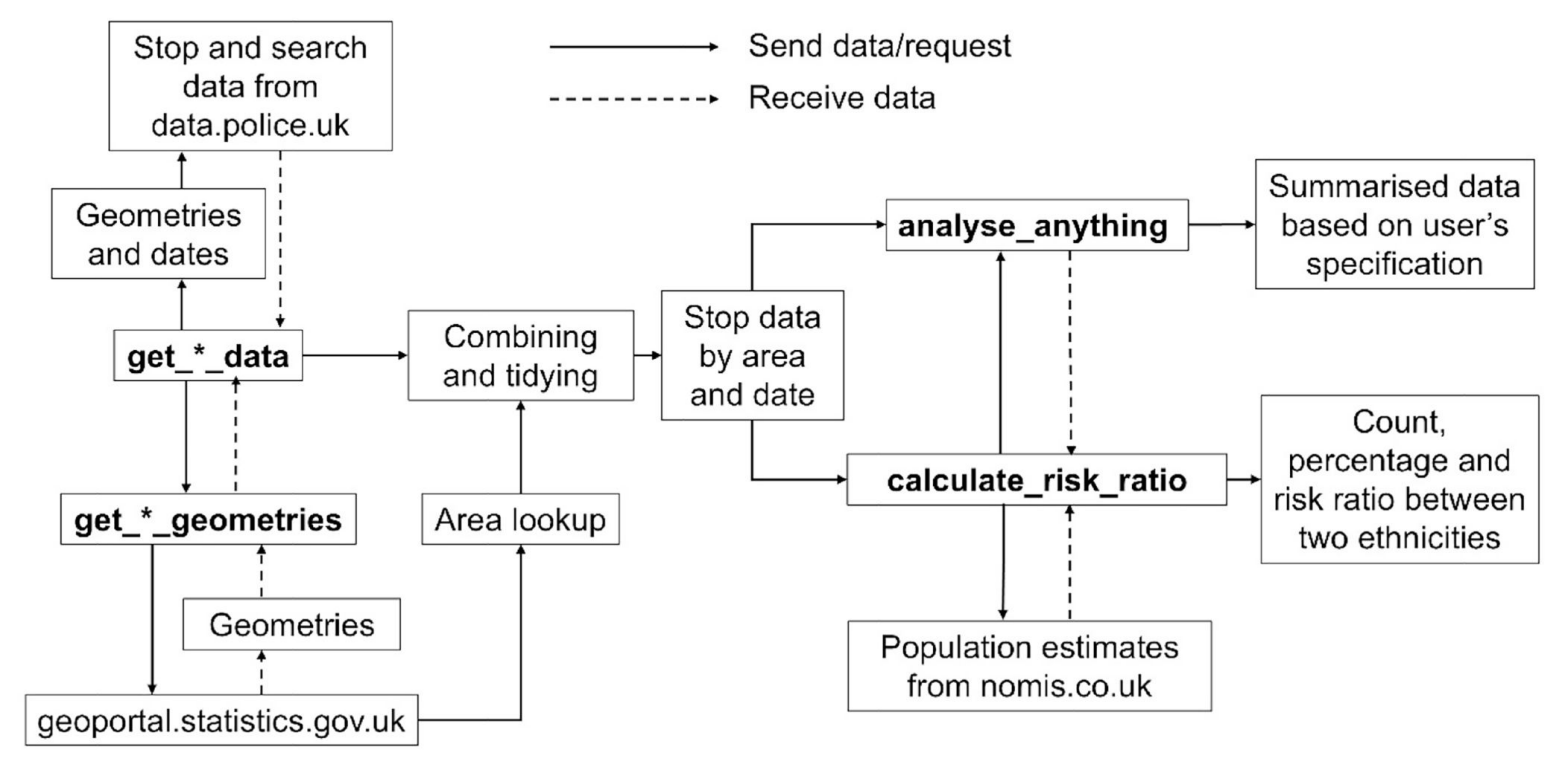
*policedatR* architecture. Bold text indicates functions within *policedatR*. Solid arrows indicate information sent to an API or another function; dashed arrows indicate information received from an API or another function

The *policedatR* package streamlines the acquisition of stop and search data across England and Wales by automating iterative HTTP requests to the data.police.uk API and enriching the results with geographic identifiers and population estimates. The package’s data acquisition functions enable efficient data collection at multiple geographic scales, generating clean, analysis-ready datasets that would otherwise require thousands of individual API calls. The analytical functions provide accessible tools for descriptive analysis and ethnic disparity calculations and are particularly useful for exploratory work and policy engagement. An overview of the package architecture is visualised in Fig. 1.

### HTTP requests

Requests to the data.police.uk API involve two parameters: ‘poly’ and ‘date’. The ‘date’ argument is the month and year of interest. The ‘poly’ argument is a set of longitudes and latitudes that describe a polygon within which to return stop records. The result of the request is a list of the stop and search records meeting the specified criteria in JavaScript Object Notation (JSON).

This HTTP request needs to be repeated for each area and date for which stop and search records are desired. Thus, to acquire stop and search records for each of the 331 Local Authority Districts (LADs) in England and Wales over the course of 12 months, a user would need to make 3972 separate requests. This number is much larger for smaller areas such as Middle layer Super Output Areas (MSOAs) and below.

*policedatR* addresses these challenges by programmatically acquiring ‘geometries’ (variables describing geographic areas using strings of longitude-latitude pairs) from the Office for National Statistics (ONS) GeoPortal (https://geoportal.statistics.gov.uk/) and passing them iteratively alongside date specifications to the data.police. uk API. The responses from the API are collated into a single dataset, which is enhanced with additional geographic detail and Census 2021 population estimates acquired from the ONS’ NOMIS API (https://www.nomisweb.co.uk/*)*, which can be used to calculate stop rates and risk ratios.

### Using *policedatR* – core functions

#### get_*_data

The ‘get_*_data’ family of functions retrieves stop data by region, PFA, LAD, MSOA, LSOA, and Output Area (OA) and collates it in a data frame. The arguments for these functions are laid out in Supplementary Table 1. The most important argument is the ‘subset’ argument, which specifies the areas for which data are desired. For example, ‘*get_lad_data(subset = list(‘lad22nm’ = Haringey))*’ acquires stops for the local authority Haringey; ‘*get_lad_data(subset = list(‘lad22nm’ = c(Haringey, Hackney))*’ acquires data for both Haringey and Hackney. If unspecified, the function will acquire data for all areas e.g. ‘*get_region_data()’* would acquire data for all regions.

The data.police.uk API uses a ‘leaky bucket’ algorithm for call limits; at the time of writing this is set to an average of 15 requests per second, *with up to 30 requests in a single second*.^5^ In the event a request fails (e.g. because the server times out), *‘get_data’* will provide an error code, wait a number of seconds and retry up to a set number of maximum tries. The number of seconds it waits and the maximum number of retries (‘wait_time’ and‘max_tries’, respectively) can be set by the user. It is possible for no stops to be found for some area-period combinations. A flag variable ‘stops_found’ explicitly tags cases where no stops are found(‘*stops_found = FALSE*’); values for other variables in these cases will return NA.

#### analyse_anything

The ‘*analyse_anything*’ function calculates descriptive statistics of stop data acquired using *policedatR* for any combination of variables present in the data. All arguments for ‘*analyse_anything’* are detailed in Supplementary Table 2. Its key argument ‘*analysis_variables*’ specifies an ordered set of grouping variables to be passed to ‘*dplyr::group_by*’ and subsequently ‘*dplyr::summarise*’ (Wickham et al., 2025), whereupon stop records are counted according to the grouping variables and then expressed as a percentage of the total count, up to the penultimate grouping variable. For example, ‘*analysis_variables = c(“area”, “period”, “ethnicity”)*’ will produce counts for each area-period-ethnicity combination, with each count expressed as a percentage of the total count for each area-period combination. Thus, in this case, each percentage describes ‘the percentage of stops in area A and time period B that are of people of ethnicity C.’ The ‘*analysis_variables*’ argument provides versatility to the function; any combination of variables can be requested. A helper function called ‘*show_analysis_variables()*’ describes the variables that can be used (detailed in Supplementary Table 3). However, users should ensure that combinations are conceptually meaningful.

The *‘period*’ argument defines the number of months each time period should be. For example, ‘*period = 2*’ produces bi-monthly counts, and ‘*period = 3*’ produces quarterly counts. The function checks whether the input value of ‘*period*’ is a factor of the number of months in the data and warns the user if it is not. If ethnicity is specified in ‘*analysis_variables*’, the function will combine the stop counts with Census 2021 population estimates by ethnicity acquired from the ONS NOMIS API and produce stop rates per 1000 population for each ethnicity. The exact ethnic groups acquired depends on the specification of ‘*ethnicity_definition*’ and ‘*collapse_ethnicity*’. If ‘*ethnicity_definition == ‘self ‘*and ‘*collapse_ethnicity = = FALSE*’, the Census 2021 19 + 1 classification is used; otherwise, the ethnicities are mapped to the 5 + 1 classification *(A Technical Guide to Ethnicity and the Criminal Justice System, 2022*, 2024; *Ethnic Group Classifications: Census 2021 - Office for National Statistics*, 2023*)*.

#### calculate_riskratio

‘*calculate_riskratio*’ takes ‘*analyse_anything*’ a step further to estimate the disparity in stop rates between two ethnicities specified by the user using the ‘*comparison*’ argument. This is a character vector of length 2. Order is important; the first element in the vector is the reference category, and the second element is the test category to which the risk ratio applies. By default, ‘*calculate_risk-ratio*’ first calls ‘*analyse_anything*’ with ‘*analysis_variables = c(“area”, “period”, “ethnicity”)*’ to get stops by ethnicity for each area-period combination. The user can omit either or both of area and period to analyse across place and time, respectively. The stop rates for each ethnicity produced by ‘*analyse_anything*’ are then compared using ‘*epitools::riskratio*’ (Aragon, 2004*)* to produce a risk ratio and 95% confidence interval (Wald approximation) and Fisher’s exact test for p-values (to account for the possibility of small cell counts for some comparisons). All arguments for ‘*calculate_riskratio*’ are detailed in Supplementary Table 4.

### Example analyses

In this section we walk through examples of how *police-datR* can be used to extract and analyse stop and search data. The code examples assume that *policedatR* has already been installed per the instructions in the package README.^6^ In this exploration we will first look at the total number of stops across England and Wales and explore variations in the proportion of controlled drugs searches across police forces. In the second example we look at overall disproportionality and variations across police forces. Example three takes this further by looking at MSOA level disproportionality in a local authority. Finally, example four provides a demonstration of how data acquired using policedatR can be combined with other datasets (in this case the Indices of Deprivation; Ministry of Housing, Communities and Local Government, 2025) to explore broader questions relating to stop and search.

#### Example 1: Stops and objects of search over time

To explore stops and objects of search over time we can acquire data for the whole of England and Wales and calculate a range of descriptive statistics. Here we use `*get_ pfa_data*’ with the default options to acquire data for all Police Force Areas (PFAs) going back 12 months from the most recent date available.^7^ In this example, the period is the 12 months to 1 April 2025.


pfa_data <– policedatR::get_pfa_data()
#	You may wish to save the data to RDS for quick loading later
#	saveRDS(pfa_data, file = here (“data”,“pfa_data.rds”))


With the data in hand, we can use ‘*analyse_anything*’ to explore stops for any combination of variables. In the simplest case we can count the number of stops over the entire period.


count <– policedatR::analyse_anything(pfa_data,
analysiS–Variables = “period”)


**Fig. 2:**
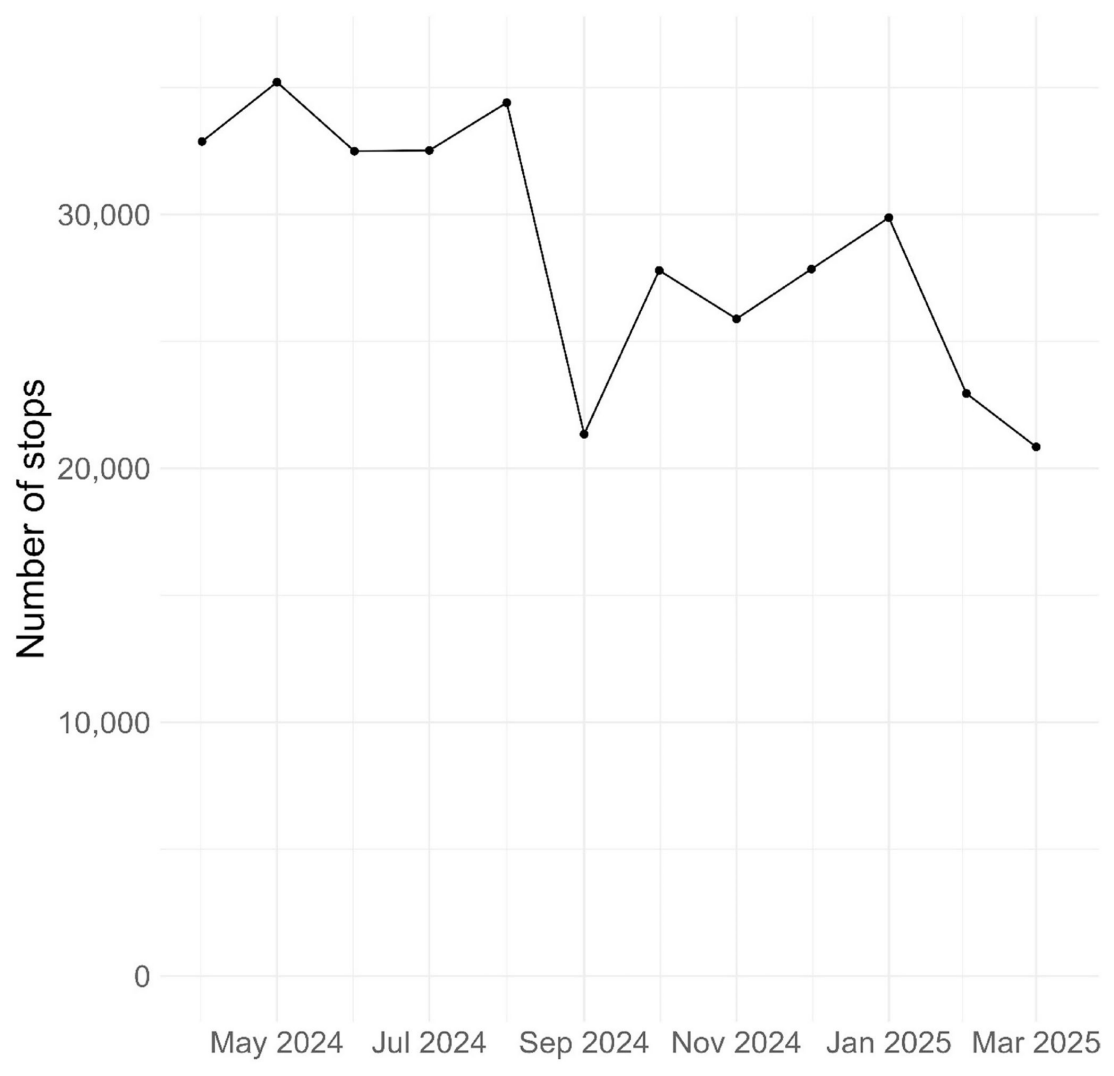
Number of stop and searches in England and Wales by month

Additionally. we can look at stops over smaller time periods. We achieve this by specifying a smaller value of ‘*period*’. Specifying ‘*period = 1*’ will produce a count for each month in the data, (Fig. 2).


count_per_month <– policedatR::analyse_anything(pfa_data,
analysis_variables = “period”, period = 1)


**Fig. 3:**
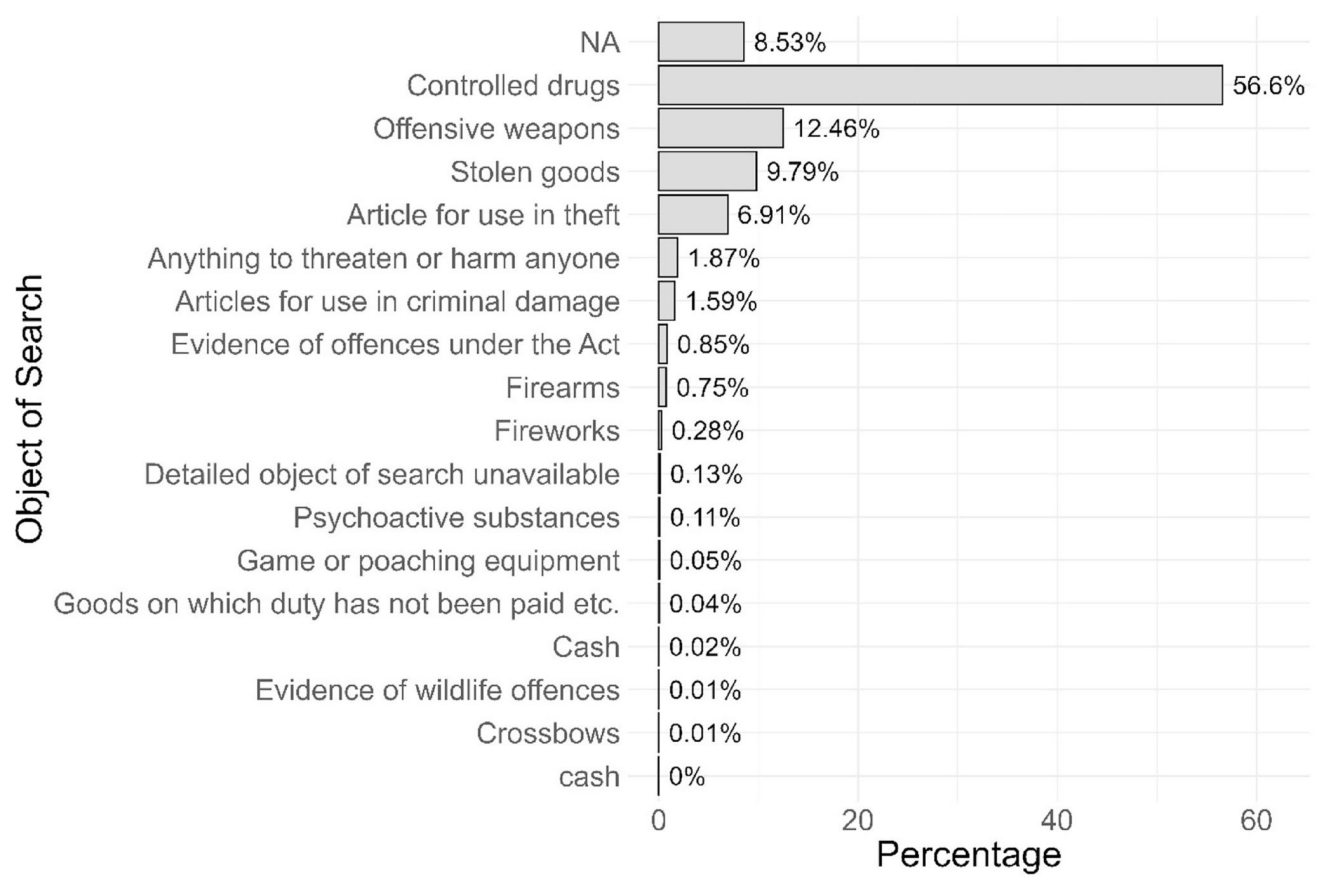
Objects of search for England and Wales 12 months to April 2025

We can also look at objects of searches across place and time (Fig. 3). This analysis indicates that most stops are for ‘Controlled drugs’ (56.6% of all stops in the period).


objects <– policedatR::analyse_anything(pfa_data,
analysis variables = c (“object”))


We can compare different areas by adding ‘area’ into the ‘*analysis_variables*’ argument. Order is important; by specifying ‘area’ first, we are saying we are interested in the percentage of stops for each object within each area.


objects_by_area <– policedatR::analyse_anything(pfa_data,
analysis_variables = c(“area”,“object”))


**Fig. 4:**
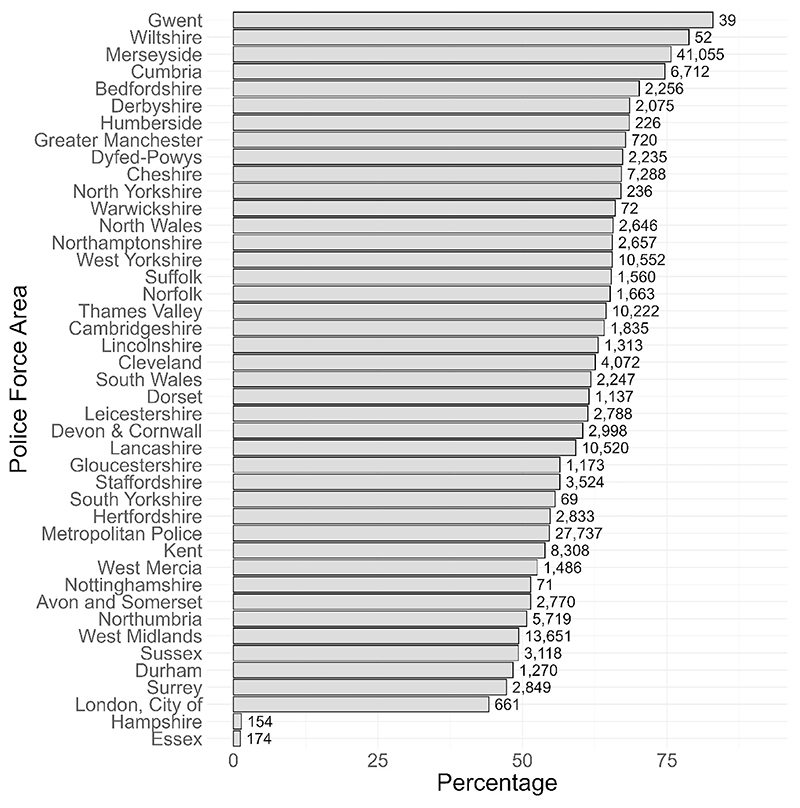
Searches for controlled drugs: 12 months to April 2025. Forces are ordered according to percentage of searches that are for drugs. Text labels refer to the raw number of stops for controlled drugs

Comparing areas for just the proportion of controlled drugs searches shows there is variation between PFAs in the extent to which stops are focused on drugs (Fig. 4). The apparently low focus on drugs in Hampshire and Essex is likely explained by the fact that *‘NA’* is recorded as the object for 77.86% of stops in Hampshire and 97.73% of stops in Essex.

This finding incidentally highlights another way in which *policedatR* may be useful; as a method for assessing the quality of the data recorded. This is important both in terms of missing entries in otherwise ‘complete’ data, as well as for understanding whether all records are actually present; the changelog at https://data.police.uk/changelog/ documents ‘known issues’ including lags in data uploads and missing data altogether due to, for example, ‘a change in IT systems.’

**Fig. 5:**
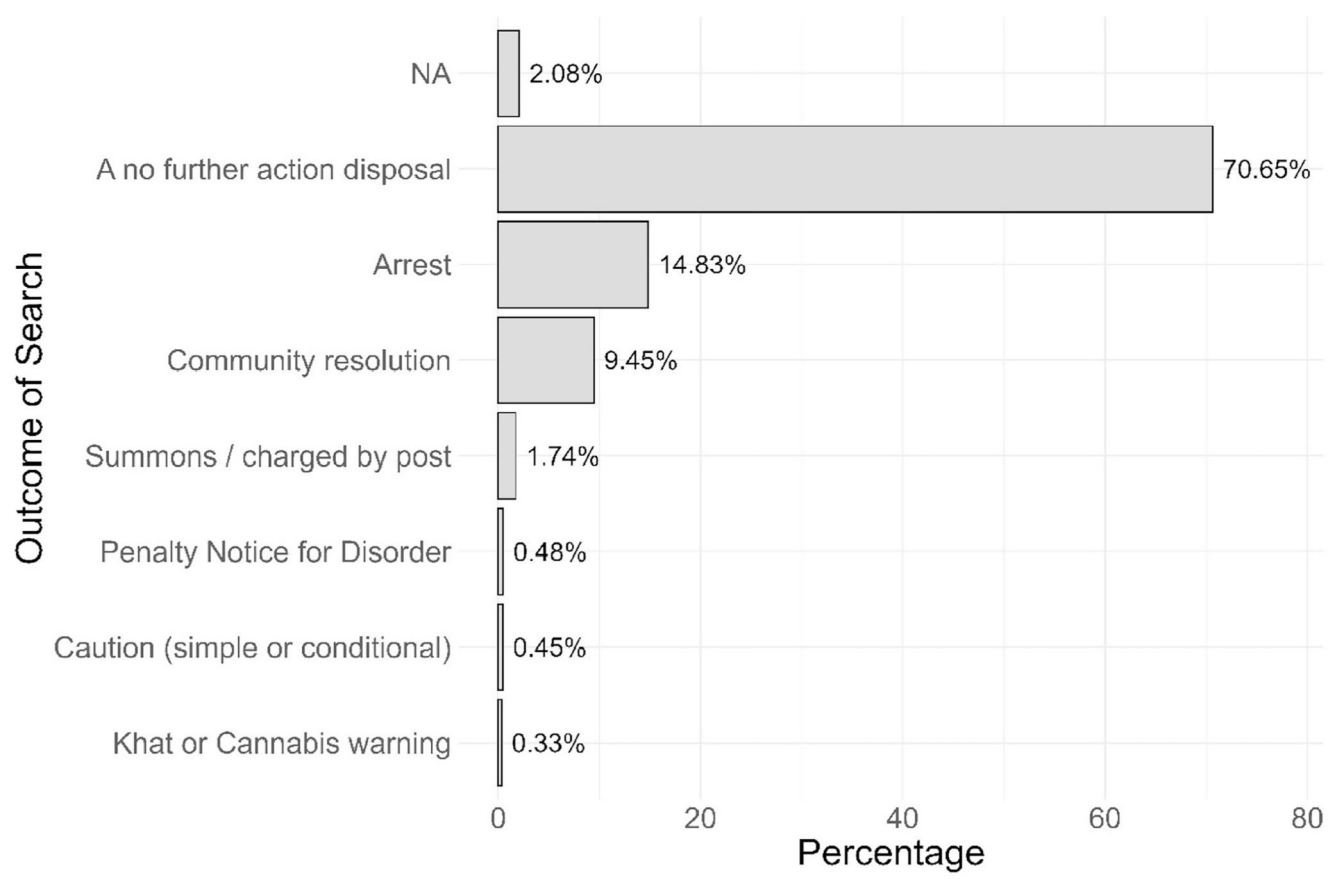
Outcome of searches: 12 months to April2025

Looking at outcomes in a similar manner, we find that across England and Wales 70.64% of searches end in a No Further Action outcome (Fig. 5).


outcomes <– policedatR::analyseanything(pfa_data,
analysis_variables = c(“outcome”))


We can combine these two analyses to focus on outcomes *within* objects by adding ‘outcome’ to ‘*analysis_variables*’. Because we are interested in the ‘outcome’ breakdown for each ‘object’, ‘object’ is listed first.


outcomes_by_object <– policedatR::analyse_anything(pfa_data,
analysis_variables = c(“object”, “outcome”))


**Fig. 6:**
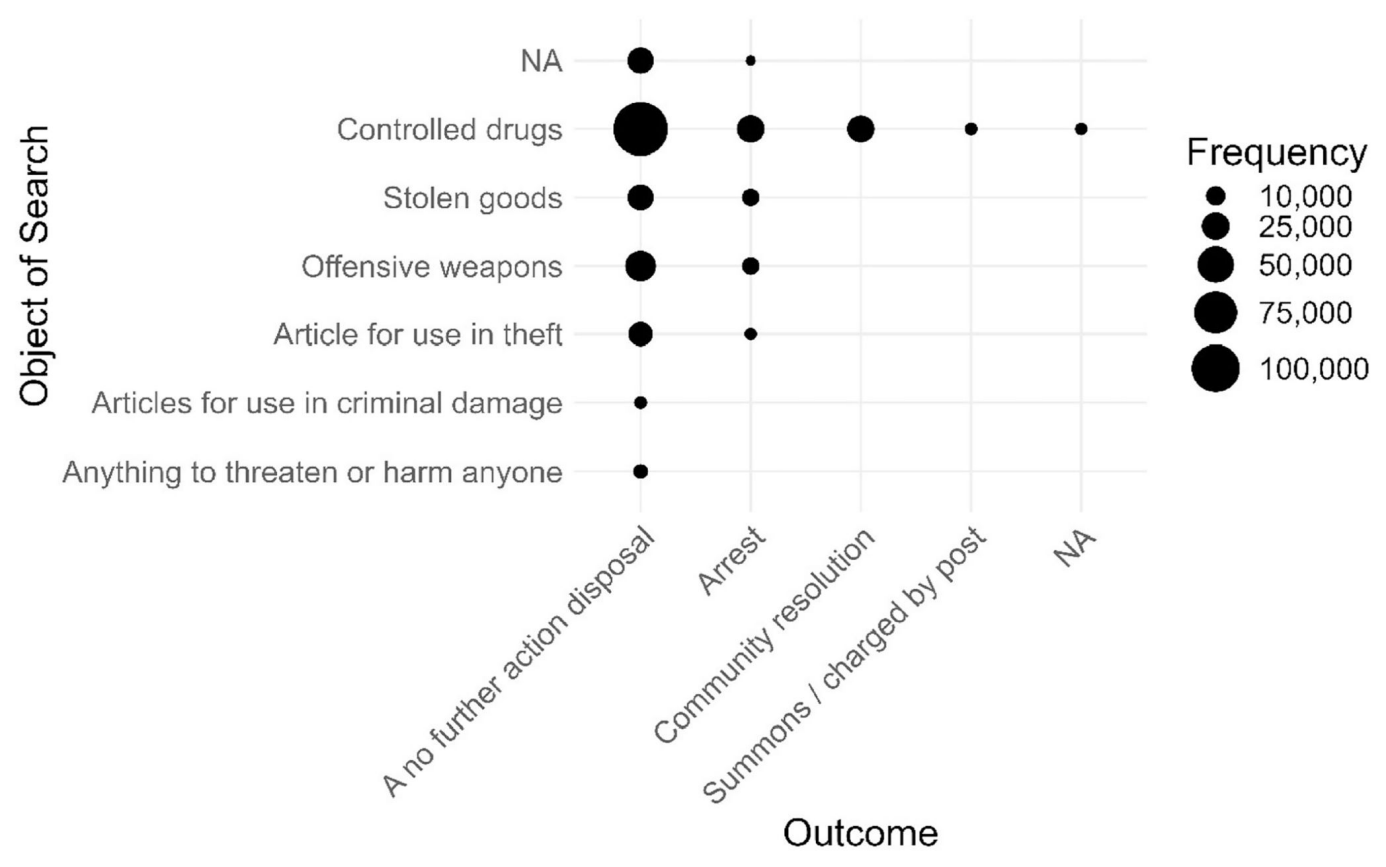
Frequency of outcomes for each object of search

As can be seen in Fig. 6, across objects of searches, most stop and search occurrences end in a no further action (NFA) disposal. For controlled drug stops which, as we have seen, account for most stops, 68.92% end in NFA. Framed differently, the ‘positive result’ rate, defined as anything that isn’t NFA, is 31.08% for drug stops.

#### Example 2: Understanding disproportionality

The ‘*calculate_riskratio*’ function produces a ratio describing the relative risk of a person of one ethnicity being stopped compared to a person of another ethnicity. We can calculate total disproportionality for the whole dataset by running ‘*calculate_riskratio*’ for each comparison of interest. Here we treat White as the reference category and run a separate comparison for each non-White ethnicity by changing the ‘*comparison*’ argument accordingly.


disp_black <–policedatR::calculate_riskratio (pfa_data, analysis_variables = “ethnicity”, ethnicity_definition = “self”, collapse_ethnicity = TRUE, comparison = c(“white”,”black”))
disp_asian <–policedatR::calculate_riskratio (pfa_data, analysiS—Variables = “ethnicity”, ethnicity_definition = “self”, collapse_ethnicity = TRUE, comparison = c(“white”,”asian”))
disp_mixed <–policedatR::calculate_riskratio (pfa_data, analysis_variables = “ethnicity”, ethnicity_definition = “self”, collapse_ethnicity = TRUE, comparison = c(“white”,”mixed”))
disp_other <–policedatR::calculate_riskratio (pfa_data, analysiS—Variables = “ethnicity”, ethnicity_definition = “self”, collapse_ethnicity = TRUE, comparison = c (“white”,”other”))


**Fig. 7:**
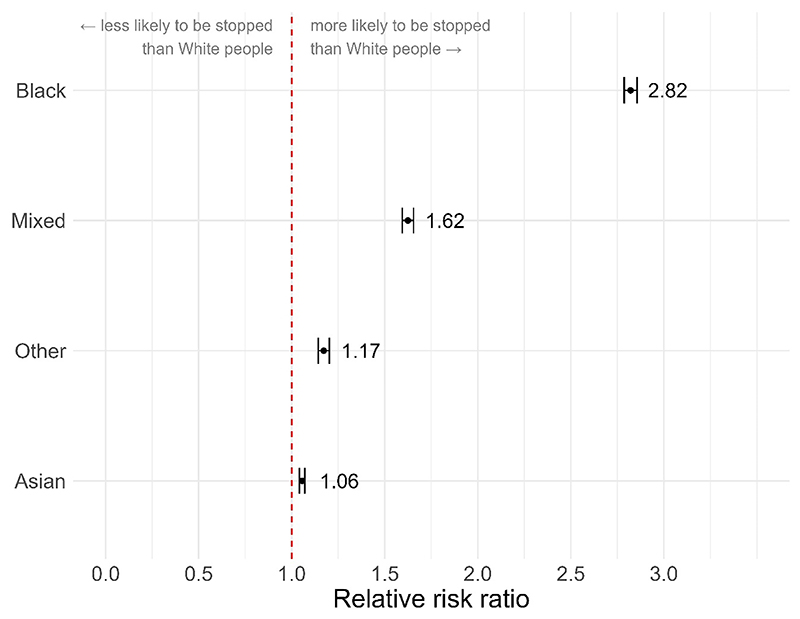
Relative risk ratio of being stopped as an ethnic minority vs. White. Dashed line indicates parity

Figure 7 shows that Black people are 2.82 times more likely than White people to be stopped and searched (*RR* = 2.82, 95% CI = [2.79–2.86], *p* < .001). People of ‘Mixed’ ethnicity are 1.62 times more likely to be stopped and searched (*RR =* 1.62, 95% CI = [1.59–1.65], *p* < .001). For people of ‘Other’ ethnicity the rate is 1.17 times (*RR* = 1.17, 95% CI = [1.14–1.2], *p* < .001) and for Asian people it is 1.06 times (*RR* = 1.06, 95% CI = [1.04–1.07], *p* < .001).

Since disproportionality is likely to vary according to differences in police practice across England and Wales, we can look at how the disparity varies by PFA. We do this by adding in ‘area’ to the ‘*analysis_variables*’ argument. For this example, we only focus on the disproportionality between Black and White people.


disp_black <– policedatR: : calculate_riskratio (pfa_data,
analysis_variables = c(“area”,”ethnicity”),
ethnicity_definition = “self”, collapse_ethnicity = TRUE,
comparison = c(“white”,”black”))


**Fig. 8:**
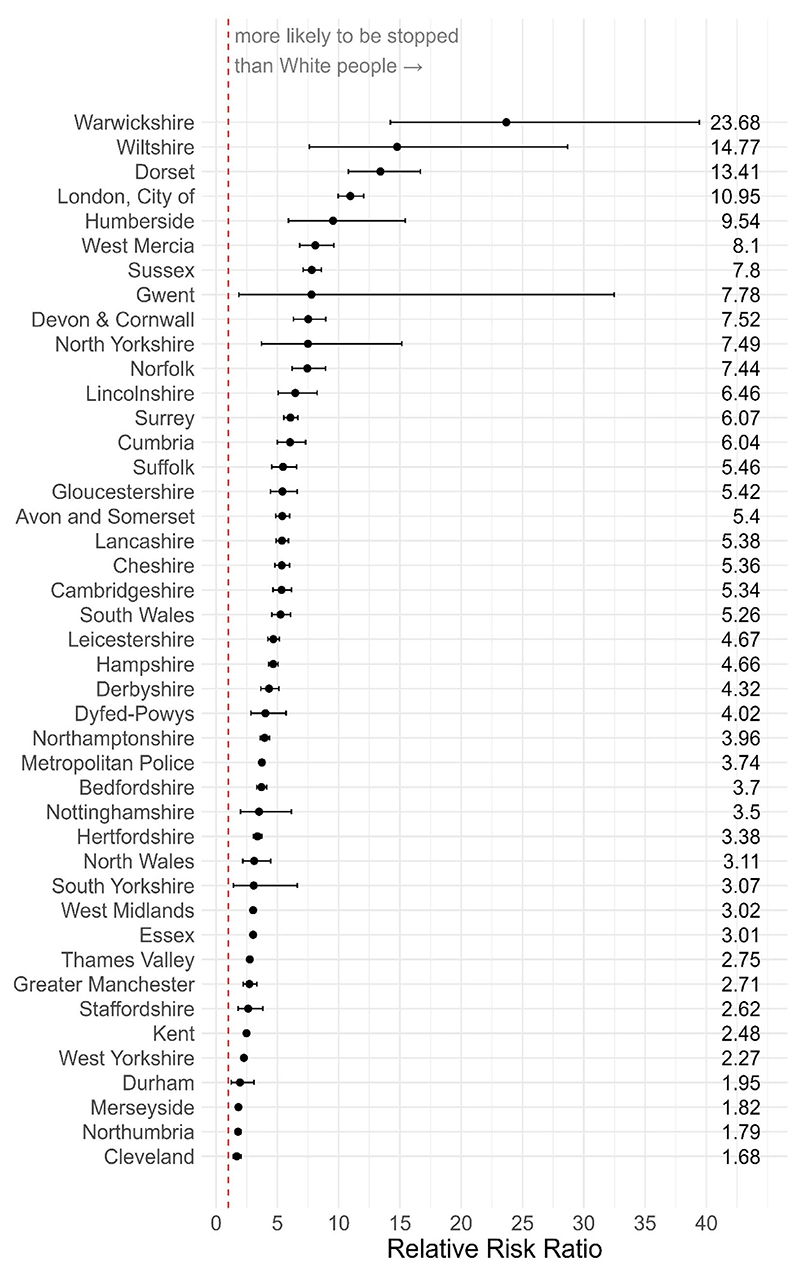
Relative risk of a Black person being stopped and searched compared to a white person across police force areas in England and Wales. Dashed line indicates parity. Forces are ordered according to risk ratio

The analysis shows there is wide variability in the extent to which Black people are disproportionately stopped and searched versus White people (though across all PFAs Black people are *more* likely to be stopped; Fig. 8).

Cleveland has the lowest disproportionality; here Black people are 1.68 times more likely to be stopped and searched than White people (*RR* = 1.68, 95% CI = [1.38–2.04], *p* < .001). Warwickshire has the highest disproportionality, where Black people are 23.68 times more likely to be stopped and searched than White people (*RR* = 23.68, 95% CI = [14.21–39.44], *p* < .001).

#### Example 3: Spatial variations in stop and search across small areas

*policedatR* makes it straightforward to explore stop rates by smaller areas. For example, we might be interested to see how stop and search varies across MSOAs within a Local Authority.

Taking the London Borough of Haringey as an example, first we extract data for the MSOAs within Haringey. This time we acquire data for the 36 months^8^ to 1 April 2025:


haringey_msoa_data <– get_msoa_data (subset = list(“lad22nm”
“Haringey”), num_months_backwards = 36)


Then we calculate disproportionality in stops between Black and White people across all time periods.


disp <– calculate_riskratio (haringey_msoa_data,
analysis_variables = c(“area”,”ethnicity”),
ethnicity_definition = “self”,collapse_ethnicity = TRUE)


For convenience it is also possible to access area geometry data via *policedatR*, making it easy to plot geospatial data. The ‘*get_msoa_geometries*’ function was in fact used in the background to query the Police API, but it can be used explicitly to get the geometries of interest.


haringey_msoa geom <–get_msoa geometries(subset =
list(“lad22nm” = “Haringey”))


**Fig. 9:**
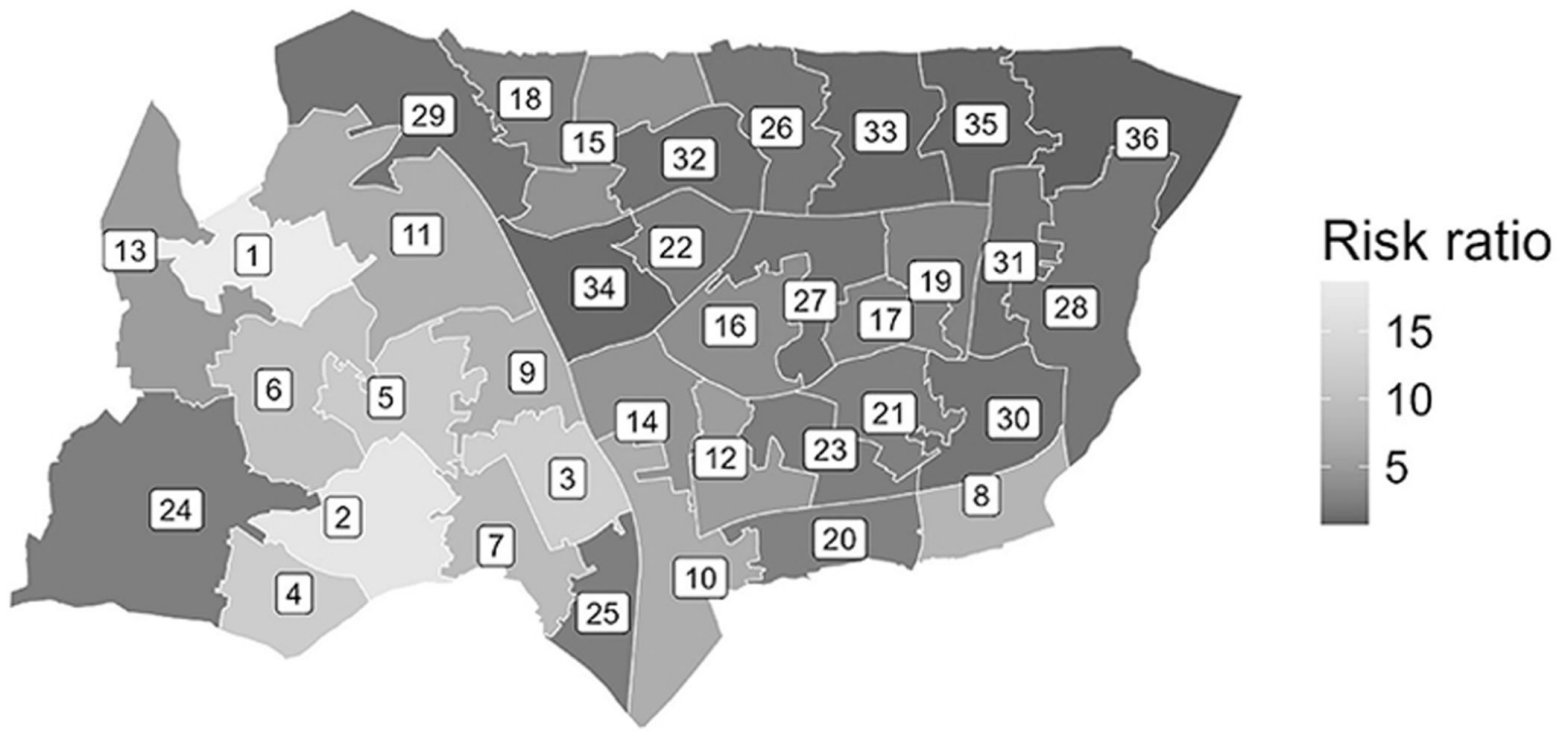
Map of haringey by rate of disproportionality

We can join the geometry data with the stop data to produce a choropleth map (Fig. 9; to be viewed alongside Table 1) showing the variation between areas in theextent to which stop and search is disproportionately targeted at Black people versus White people. The results indicate consistent but varied disproportionality in Haringey, with Black people being between 1.2 and 22.04 times more likely to be stopped than White people. It is important to note that at this level of analysis risk ratios can be very large, with wide confidence intervals. This is often due to small population denominators in these areas (e.g. in this case, small Black populations) leading to inflated point estimates. Consequently, these estimates should be interpreted alongside the count data and confidence intervals, and *policedatR* produces these values in the same data frame as the risk ratio.

#### Example 4: Combining stop and search data with other data

A key advantage of data acquired using *policedatR* is that it is straightforward to combine with other datasets organised by geography to explore broader questions around policing. Here we demonstrate this by combining stop and search data across London at MSOA level with Indices of Deprivation (IoD) data (Ministry of Housing, Communities and Local Government, 2025; Noble et al., 2025). We retrieved MSOA-level stop data for London for the 12 months to 30 September 2025 (inclusive) and used the ‘*analyse_anything*’ function to produce stop counts for each MSOA in London. We then joined this data with IoD MSOA-level data.


# Get the loD data
url <– ‘https://github.com/JustKnowledge-UK/public-downloads/releases/download/data-release/iod25_msoa.csv’
filename <– basename(url)
filepath <– here(“data”, filename)
download.file(url, destfile =filepath, mode = “wb”)
iod25_msoa <– read_csv(filepath)
#	Get the Stop and Search data
london_msoa_data <– get_msoa_data(subset = list(“rgn22nm”
“London”), num_months_backwards = 12)
#	Produce counts
london msoa summary <– analyseanything(londonmsoadata,
analysis_variables = “area”)
#	Join the data
joined_data <– london_msoa_summary %>%
left_join(iod25_msoa, by = ‘msoa21cd’)


**Fig. 10:**
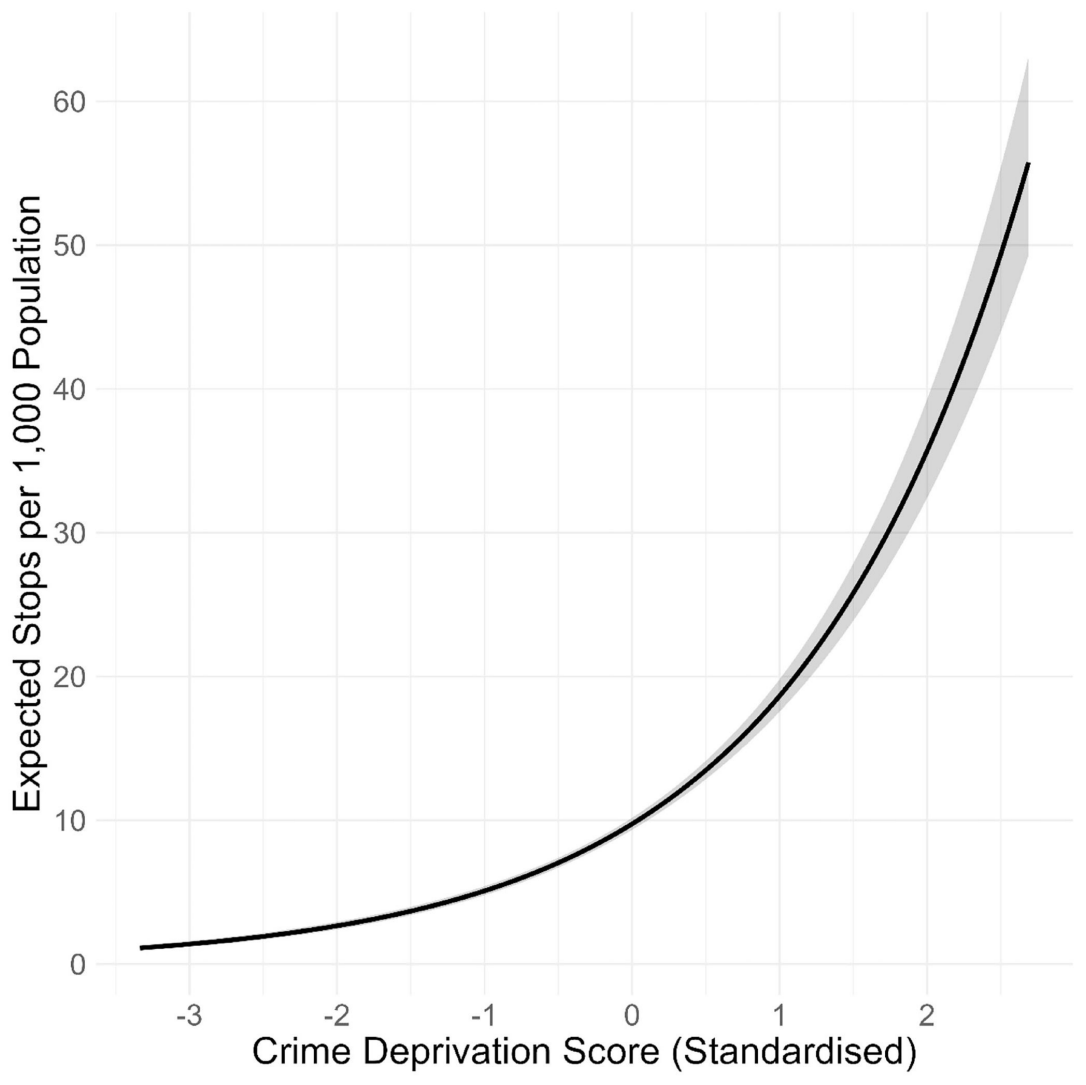
Fixed effect of crime deprivation on stop rate. Shaded area shows 95% confidence interval

A key question concerns how stop and search practice relates to patterns of crime. We explored this by testing the relationship between crime deprivation score (standardised) and stop and search counts using a Poisson mixed effects model with a random intercept for each MSOA to account for unobserved heterogeneity and a population offset to account for exposure differences between MSOAs. Standardised crime deprivation score was a significant predictor of stop and search *(Estimate* = -0.65, *SE =* 0.02, *z* = -29.27, *p* < .001*)*. Higher (i.e., worse) crime scores were associated with higher stop and search rates; on average, a one-standard-deviation increase in crime score was associated with a 91% higher expected stop and search rate *(Incidence Rate Ratio* = 1.91, 95% Confidence Interval: 1.83-2.00; Fig. 10). This finding indicates that stop and search is deployed more strongly in areas experiencing higher burdens of crime.

## Discussion

Stop and search is a police power of major public, political and empirical interest which has hitherto been hampered by the accessibility and quality of data. The main methods of accessing stop and search data, namely Home Office annual releases, FOI requests and downloads from police.uk, are limited to varying degrees by the timeliness of publication, differences in legislative scope and geographic coverage. While the data available from data. police.uk provide the best opportunities for detailed interrogation of stop and search data, there remains a barrier insofar as specialist skills and knowledge, as well as the time spent deploying these skills, are required to most efficiently access and use the data.

The *policedatR* package resolves these issues by making data from the data.police.uk API accessible through the R programming language and systematically acquiring data for any time within a rolling 36-month period. The data also contains detailed breakdowns of the legislation used, objects and outcomes of searches, and can be acquired for a range of geographic units anywhere within England and Wales. These features open up a wide range of research opportunities on stop and search.

We have illustrated how *policedatR* can be used to investigate temporal trends in stop and search, explore the objects and outcomes of searches, make comparisons between different areas, and quantify racial disparities across time and space. We have also demonstrated how data acquired using policedatR can be joined with other data sources to explore stop and search in relation to other factors. Many other analyses are possible; the geographic units implemented by *policedatR* make stop and search data immediately compatible with any data collected by Census geography. This versatility will enable researchers to explore, with greater ease and precision, nuanced questions around stop and search. We believe this has the potential to support efforts for more equitable policing by making acquisition of police data simpler and at the same time enhancing the data to make it more amenable to temporal and spatial analyses and harmonisation with other data.

Moreover, stop and search may be an important ecological factor in research on other subjects, especially those focusing on racialised populations such as community health outcomes, educational attainment patterns, or local economic development, where policing practices (and inequalities therein) may serve as important context that influences or correlates with these broader social phenomena. *policedatR* makes such analyses straightforward and manageable for researchers.

### Limitations

Data is uploaded to data.police.uk by each police force monthly, but forces differ in the consistency with which data is uploaded and there are occasions when no data are uploaded by a force for months at a time due to a range of issues. While we plan to update *policedatR* with functions to help identify and quantify missingness, for now there should be no assumption that very recent data is complete. Data.police.uk maintains a changelog detailing which data have not been submitted and, in some cases, where data has been ‘refreshed’. Users of police-datR (and police.uk generally) should refer to the changelog and, where relevant, report missingness and counts alongside risk ratios in empirical work that relies on these data.

As is the case for most statistics on stop and search, *policedatR* uses resident population estimates as denominators in the calculation of stop rates. We therefore assume that resident populations and their demographics roughly approximate those of the people ‘available’ to be stopped in areas. However, because stops are made in public places, there is no guarantee that the demographic profile of the residents of an area matches the profile of people using the public spaces in that area. Furthermore, areas differ in the footfall they receive, with urban centres seeing higher concentrations of people than suburbs and rural areas. Using the resident population as the denominator for stop rates cannot account for the movement of people or the variation in footfall between areas and it has consequently been criticised as not reflecting the ‘operational context’ of policing (Ratcliffe & Hyland, 2025). Nonetheless, alternatives are limited and have their own limitations, and some suggest the resident population remains the best option (Quinton, 2024). However, since *policedatR* provides the underlying raw data, researchers can implement their own denominators (e.g. daytime populations, denominators derived from mobility data) straightforwardly.

## Conclusion

Stop and search represents one of the most contentious police powers in England and Wales, with profound implications for police-community relations, social justice, and public policy. Despite its significance, empirical research on stop and search in England and Wales has been constrained by substantial barriers to data access.

The *policedatR* package addresses these challenges by providing researchers, policymakers, and civil society organisations with streamlined access to comprehensive stop and search data across England and Wales. By automating the complex process of data acquisition from the data.police.uk API and enriching these data with geographic identifiers and population estimates, *police-datR* transforms what previously required thousands of individual API calls and extensive data carpentry into a straightforward analytical workflow.

Our demonstration analyses illustrate the package’s capacity to facilitate sophisticated research questions that would have been prohibitively time-consuming using traditional data access methods. The ability to seamlessly examine temporal trends, geographic variations, and ethnic disparities at multiple spatial scales opens new possibilities for understanding the nuanced deployment of stop and search powers.

Beyond academic research, *policedatR* has implications for public engagement with policing practices. By making stop and search data more accessible, the package enables civil society organisations, journalists, and community groups to conduct their own analyses and supports their ability to hold police forces accountable for their use of these powers. The package’s future data quality auditing capabilities may also provide a mechanism for identifying and addressing gaps in police data provision, potentially improving the overall transparency of policing practices. The *policedatR* package provides a contribution to the analytical infrastructure necessary for rigorous, evidence-based evaluation of police practices which we hope will serve not only academic research but also broader public interest and engagement in fair and effective policing.

## Supplementary Material

The online version contains supplementary material available at https://doi.org/10.1186/s40163-025-00266-6.

Supplementary Information

## Figures and Tables

**Table 1:** Risk ratios and confidence intervals corresponding to places in Fig. 9

Index	Area name	Risk ratio	95% confidence interval	*P*
1	Haringey 014	22.04	[10.01−48.53]	< 0.001***
2	Haringey 033	18.87	[8.33−42.74]	< 0.001***
3	Haringey 035	12.76	[6.15−26.46]	< 0.001***
4	Haringey 022	12.42	[7.79−19.82]	< 0.001***
5	Haringey 034	10.69	[6.29−18.18]	< 0.001***
6	Haringey 028	10.06	[3.3−30.66]	< 0.001***
7	Haringey 021	9.46	[5.24−17.08]	< 0.001***
8	Haringey 029	8.43	[6.39−11.12]	< 0.001***
9	Haringey 020	7.88	[4.88−12.74]	< 0.001***
10	Haringey 031	7.63	[6.15−9.46]	< 0.001***
11	Haringey 009	7.52	[4.86−11.62]	< 0.001***
12	Haringey 027	5.61	[3.68−8.57]	< 0.001***
13	Haringey 017	5.60	[2.27−13.8]	0.001***
14	Haringey 023	4.40	[3.54−5.48]	< 0.001***
15	Haringey 019	3.95	[2.52−6.19]	< 0.001***
16	Haringey 007	3.88	[2.95−5.11]	< 0.001***
17	Haringey 036	3.02	[1.64−5.56]	0.001***
18	Haringey 011	2.92	[2.12−4.01]	< 0.001***
19	Haringey 018	2.82	[1.66−4.79]	< 0.001***
20	Haringey 024	2.81	[2.42−3.26]	< 0.001***
21	Haringey 032	2.71	[1.78−4.14]	< 0.001***
22	Haringey 010	2.65	[1.8−3.9]	< 0.001***
23	Haringey 030	2.44	[1.09−5.45]	0.036*
24	Haringey 001	2.40	[1.46−3.96]	0.001***
25	Haringey 026	2.35	[1.75−3.15]	< 0.001***
26	Haringey 005	1.95	[1.59−2.37]	< 0.001***
27	Haringey 015	1.89	[1.63−2.2]	< 0.001***
28	Haringey 013	1.88	[1.08−3.24]	0.03*
29	Haringey 004	1.85	[1.4−2.44]	< 0.001***
30	Haringey 025	1.83	[1.57−2.13]	< 0.001***
31	Haringey 012	1.70	[1.31−2.19]	< 0.001***
32	Haringey 008	1.65	[1.33−2.04]	< 0.001***
33	Haringey 006	1.27	[0.92−1.74]	0.151
34	Haringey 016	1.20	[1.03−1.39]	0.021*
35	Haringey 037	1.09	[0.95−1.24]	0.252
36	Haringey 002	0.79	[0.6−1.03]	0.078

## Data Availability

The data and scripts used to produce the example analysis in this paper can be found in the Open Science Framework repository at https://osf.io/sj376/?view_only=cbbe71767ab342648fdb9015b6fa87db. The data were obtained via *policedatR* from the API at data.police.uk and are licenced under the Open Government Licence v3.0. Specifically, three datasets were created using the policedatR pipeline: 1. Stop and searches in England and Wales for theperiod 1 April 2024 (inclusive) to 1 April 2025 (exclusive), broken down by Police Force Area. In this dataset each row represents an individual stop record. 2. Stop and searches in the London Borough of Haringey for the period 1 April 2022 (inclusive) to 1 April 2025 (exclusive), broken down by Middle layer Super Output Area. In this dataset each row represents an individual stop record. 3. Stop and searches for the whole of London for the period 1 October 2024 (inclusive) to 1 October 2025 (exclusive), broken down by Middle layer Super Output Area. In this dataset each row represents an individual stop record. We also produced a harmonised dataset, combining the summarised data from 3 above with English Indices of Deprivation data (2025; also licenced under Open Government Licence v3.0 and acquired from https://www.gov.uk/government/statistics/english-indices-of-deprivation-2025) aggregated to Middle layer Super Output Area. In this dataset, each row represents a Middle layer Super Output Area. See the policedatR GitHub repository for installation and usage instructions for the package itself: https://github.com/JustKnowledge-UK/policedatR.

## Declarations

1 Policing in the UK is devolved. In this paper we focus on the England and Wales context.

2 police.uk provides local policing information and performance data across England and Wales.

3 See https://www.police.uk/ and https://data.police.uk/.

4 [Redacted to preserve anonymity].

5 *See* https://data.police.uk/docs/api-call-limits/ *for more information*.

6 The README – and the code and data used to generate this example analysis – can be found in the Open Science Framework repository (https://osf.io/sj376/?view_only=cbbe71767ab342648fdb9015b6fa87db.

7 Note that this can be time consuming at ~ 2 h.

8 *Any number of months backwards can be specified up to a maximum of 36. This limit is imposed by the API at data.police.uk. Data older than 36-months is archived at data.police.uk and remain available for manual download*.
