# Supplementary material for: policedatR: a comprehensive R package for stop and search data in England and Wales
Source: Crime Sci. Author manuscript; Available in PMC 2026 May 6. (PMC13099712; doi:10.1186/s40163-025-00266-6)
Supplement: Supplementary Information [file EMS213290-supplement-Supplementary_Information.docx]

**Supplementary Information**

**Article title:** policedatR – A comprehensive R package for Stop and Search Data in England and Wales

**Journal:** Crime Science

**Authors:** Jolyon Miles-Wilson^1^ and Celestin Okoroji^1,2^

**Affiliation:** *1* Just Knowledge, incubated by Ratio Research Community Interest Company, 7 Bell Yard, London, WC2A 2JR*; 2* London School of Economics and Political Science, Houghton St, London WC2A 2AE

**Correspondence:** [jolyon.miles-wilson@justknowledge.org.uk](mailto:jolyon.miles-wilson@justknowledge.org.uk)

**Supplementary Tables**

Table 1 get_*_data arguments

| **Argument** | **Description** | **Format** | **Default** |
| --- | --- | --- | --- |
| subset | A named list defining the areas for which data are required. Names correspond to the area variable on which to subset. Values correspond to the desired values of the area variable. If NULL will acquire for all areas. | Named list (e.g., list(‘lad22nm’ = ‘Haringey’)) | None |
| num_months_backwards | The number of months backwards from the start point for which to acquire data. Maximum is 36 (limit imposed by API). | Numeric | 12 |
| oldest_month | Instead of specifying num_months_backwards, the user can specify the oldest month of interest (in combination with the oldest year of interest). | Numeric (e.g. 8 for August) | None |
| oldest_year | Instead of specifying num_months_backwards, the user can specify the oldest year of interest (in combination with the oldest month of interest). | Numeric (e.g. 2024) | None |
| most_recent_month | The most recent month of interest By default the function will determine this based on the most recent API update. | Numeric (e.g. 8 for August) | Most recent as determined by API |
| most_recent_year | The most recent month of interest. By default the function will determine this based on the most recent API update. Numeric value in format YYYY (e.g., 2021). | Numeric (e.g. 2024) | Most recent as determined by API |
| wait_time | If there is a server error when submitting POST request, the function will wait wait_time seconds before retrying. | Numeric | 5 |
| max_tries | The maximum number of times to try a POST request. | Numeric | 10 |
| include_no_stop_areas | Whether to include areas for which there are no results. Default is TRUE (recommended as this will flag areas for which there are no stops and so provide most comprehensive picture). | Boolean | TRUE |

Table 2 analyse_anything arguments

| **Argument** | **Description** | **Format** | **Default** |
| --- | --- | --- | --- |
| data | The stop records to analyse. | Tibble or data frame acquired using policedatR | None |
| analysis_variables | A character vector of variables to be used as grouping variables when counting. Order is important. Percentage denominators are the sum of counts within the final grouping level. Use *show_analysis_variables()* to see these values and brief explanation of to what they refer | Character vector (e.g., c(“object”, “outcome”) | None |
| ethnicity_definition | If ethnicity is included, what definition to use. 'self' for self-defined, 'officer' for officer-defined. | Character | None |
| collapse_ethnicity | If ethnicity is included, should it be aggregated (TRUE) or not (FALSE)? If ethnicity_definition == 'self', this controls whether to use the 19 disaggregated categories or to aggregate to the 5 broader categories. If ethnicity_definition == 'officer', collapse_ethnicity is always TRUE. | Boolean | None |
| period | The number of months to use as a time period, e.g., 6 = 6-month-periods; 1 = monthly periods. | Numeric | 12 |

Table 3 analysis_variables options

| **Variable** | **Description** |
| --- | --- |
| Age | The age range of the person stopped |
| Ethnicity | The ethnicity of the person stopped (follows definition and aggregation specified by user) |
| Gender | The gender of the person stopped |
| Area | The geographic area, e.g. local authority. Inherits from data. |
| Object | The item the Police report was being sought |
| Legislation | The legislation used to make the stop |
| Outcome | The result of the stop and search |
| Period | The time period as specified in the analysis function call |

Table 4 calculate_riskratio arguments

| **Argument** | **Description** | **Format** | **Default** |
| --- | --- | --- | --- |
| data | The stop records to analyse. | A tibble or data frame acquired using policedatR | None |
| ethnicity_definition | Which ethnicity definition to use for counts. 'self' has the possibility of using Census 2021 19 disaggregated categories. 'officer' is the Census 2021 5 aggregated ethnicity categories. | Character | None |
| collapse_ethnicity | If ethnicity is included, should it be aggregated (TRUE) or not (FALSE)? If ethnicity_definition == 'self', this controls whether to use the 19 disaggregated categories or to aggregate to the 5 broader categories. If ethnicity_definition == 'officer', collapse_ethnicity is always TRUE. | Boolean | None |
| comparison | The ethnicities to compare. A character vector with length 2, where the first element is the reference level of ethnicity against which the second ('test') level is compared. If NULL, user will be prompted to pick the reference and test ethnicities. | Character vector of length 2 | *c(“white”, “black”)* |
| period | The number of months to use as a time period, e.g., 6 = 6-month-periods; 1 = monthly periods. | Numeric | None |
